# Assessment of Health Care Exposures and Outcomes in Adult Patients With Sepsis and Septic Shock

**DOI:** 10.1001/jamanetworkopen.2020.6004

**Published:** 2020-07-07

**Authors:** Katherine Fay, Mathew R. P. Sapiano, Runa Gokhale, Raymund Dantes, Nicola Thompson, David E. Katz, Susan M. Ray, Lucy E. Wilson, Rebecca Perlmutter, Joelle Nadle, Deborah Godine, Linda Frank, Geoff Brousseau, Helen Johnston, Wendy Bamberg, Ghinwa Dumyati, Deborah Nelson, Ruth Lynfield, Malini DeSilva, Marion Kainer, Alexia Zhang, Valerie Ocampo, Monika Samper, Rebecca Pierce, Lourdes Irizarry, Marla Sievers, Meghan Maloney, Anthony Fiore, Shelley S. Magill, Lauren Epstein

**Affiliations:** 1 Division of Bacterial Diseases, National Center for Emerging and Zoonotic Infectious Diseases, Centers for Disease Control and Prevention, Atlanta, Georgia; 2 Epidemic Intelligence Service, Centers for Disease Control and Prevention, Atlanta, Georgia; 3 Division of Healthcare Quality Promotion, National Center for Emerging and Zoonotic Infectious Diseases, Centers for Disease Control and Prevention, Atlanta, Georgia; 4 Emory University School of Medicine, Atlanta, Georgia; 5 Georgia Emerging Infections Program, Decatur; 6 Maryland Department of Health, Baltimore; 7 California Emerging Infections Program, Oakland; 8 Colorado Department of Public Health and Environment, Denver; 9 New York Emerging Infections Program, University of Rochester Medical Center, Rochester; 10 Minnesota Department of Health, St Paul; 11 Tennessee Department of Health, Nashville; 12 Oregon Health Authority, Portland; 13 New Mexico Department of Health, Santa Fe; 14 Connecticut Emerging Infections Program, Hartford

## Abstract

**Importance:**

Current information on the characteristics of patients who develop sepsis may help in identifying opportunities to improve outcomes. Most recent studies of sepsis epidemiology have focused on changes in incidence or have used administrative data sets that provided limited patient-level data.

**Objective:**

To describe sepsis epidemiology in adults.

**Design, Setting, and Participants:**

This retrospective cohort study reviewed the medical records, death certificates, and hospital discharge data of adult patients with sepsis or septic shock who were discharged from the hospital between October 1, 2014, and September 30, 2015. The convenience sample was obtained from hospitals in the Centers for Disease Control and Prevention Emerging Infections Program in 10 states (California, Colorado, Connecticut, Georgia, Maryland, Minnesota, New Mexico, New York, Oregon, and Tennessee). Patients 18 years and older with discharge diagnosis codes for severe sepsis or septic shock were randomly selected. Data were analyzed between May 1, 2018, and January 31, 2019.

**Main Outcomes and Measures:**

The population’s demographic characteristics, health care exposures, and sepsis-associated infections and pathogens were described, and risk factors for death within 30 days after sepsis diagnosis were assessed.

**Results:**

Among 1078 adult patients with sepsis (569 men [52.8%]; median age, 64 years [interquartile range, 53-75 years]), 973 patients (90.3%) were classified as having community-onset sepsis (ie, sepsis diagnosed within 3 days of hospital admission). In total, 654 patients (60.7%) had health care exposures before their hospital admission for sepsis; 260 patients (24.1%) had outpatient encounters in the 7 days before admission, and 447 patients (41.5%) received medical treatment, including antimicrobial drugs, chemotherapy, wound care, dialysis, or surgery, in the 30 days before admission. A pathogen associated with sepsis was found in 613 patients (56.9%); the most common pathogens identified were *Escherichia coli, Staphylococcus aureus, Klebsiella pneumonia*e, and *Clostridioides difficile*. After controlling for other factors, an association was found between underlying comorbidities, such as cirrhosis (odds ratio, 3.59; 95% CI, 2.03-6.32), immunosuppression (odds ratio, 2.52; 95% CI, 1.81-3.52), vascular disease (odds ratio, 1.54; 95% CI, 1.10-2.15), and 30-day mortality.

**Conclusions and Relevance:**

Most adults experienced sepsis onset outside of the hospital and had recent encounters with the health care system. A sepsis-associated pathogen was identified in more than half of patients. Future efforts to improve sepsis outcomes may benefit from examination of health maintenance practices and recent health care exposures as potential opportunities among high-risk patients.

## Key Points

**Question:**

What types of health care exposures occur during the 30 days before hospitalization of a patient with sepsis or septic shock, and how common are these exposures?

**Findings:**

In this cohort study of 1078 US adults with sepsis and septic shock across 10 states, most patients experienced sepsis onset outside of the hospital, had recent encounters with the health care system, and had a sepsis-associated pathogen documented; 42% of patients received antimicrobial drugs, chemotherapy, wound care, dialysis, or surgery in the 30 days before sepsis occurred. After controlling for other factors, an association was found between underlying comorbidities, such as cirrhosis, immunosuppression, and vascular disease, and 30-day mortality.

**Meaning:**

The findings suggest that future efforts to improve outcomes among patients with sepsis and septic shock would benefit from examination of health maintenance practices and recent health care exposures as potential opportunities among high-risk patients.

## Teaser

This cohort study uses medical records, death certificates, and hospital discharge data to describe health care exposures and outcomes among adult patients with sepsis and septic shock.

## Introduction

Sepsis is a serious public health issue, with an estimated 1.7 million adult cases annually in the United States, and it is potentially associated with 270 000 deaths.^1^ Nearly 1 in 3 hospitalizations that end in death are associated with sepsis.^1^

During the last 20 years, initiatives aimed at improving sepsis outcomes have focused on protocols that emphasize early recognition and standardized treatment in a hospital.^2^ However, most sepsis cases begin outside of the hospital, encompassing diverse clinical presentations.^3,4^ Because no confirmatory test exists, the diagnosis of sepsis is based on evidence of infection, organ dysfunction, and clinical judgment. Most large-scale descriptive studies of sepsis epidemiology using administrative claims data have not included detailed medical record review and are therefore subject to several limitations.^5^

We performed detailed medical record reviews to describe the demographic and clinical characteristics, including health care exposures, pathogens, and outcomes, of persons diagnosed with sepsis and septic shock.

## Methods

We used the Emerging Infections Program (EIP), a public health surveillance and research network supported by the Centers for Disease Control and Prevention, to collect data from the medical records of patients with sepsis and septic shock.^6^ Each EIP site (California, Colorado, Connecticut, Georgia, Maryland, Minnesota, New Mexico, New York, Oregon, and Tennessee) identified 1 to 4 acute care hospitals in which to sample patients with sepsis for medical record review. Only general short-term acute care hospitals were eligible for inclusion. Eligible patients were discharged from participating hospitals between October 1, 2014, and September 30, 2015, with diagnosis codes for severe sepsis or septic shock (*International Classification of Diseases, Ninth Revision, Clinical Modification* [*ICD-9-CM*] code 995.92 or 785.52). In each EIP site, staff members randomly selected approximately 100 adult patients (aged ≥18 years) and 100 pediatric patients (aged ≤18 years) across participating hospitals for medical record review; only adult patients are included in this article. For patients with more than 1 hospitalization during the period of interest, only the first hospitalization was eligible for inclusion. We excluded patients who had no clinical documentation that specifically indicated sepsis, septic shock, or similar terms in the medical records.

The Centers for Disease Control and Prevention determined the project to be a nonresearch activity. Each EIP site and hospital review board determined the project to be a nonresearch activity or approved the project as a research activity with a waiver of informed consent. This study followed the Strengthening the Reporting of Observational Studies in Epidemiology (STROBE) reporting guideline for cohort studies.

### Data Collection

From September 1, 2017, to May 30, 2018, trained EIP staff members reviewed medical records using a standardized data collection form to abstract detailed information regarding patients’ demographic and clinical characteristics.

#### Definitions

The date of sepsis diagnosis was defined as the first date of clinical documentation by a health care practitioner of either sepsis or an associated term in the patient’s medical record. Community-onset cases were defined as cases in which the date of sepsis diagnosis occurred during the first 3 days of hospitalization (in which the date of admission was day 1). Sepsis cases for which the date of sepsis diagnosis occurred after day 3 of hospitalization were classified as hospital-onset cases.

We defined prehospital medical treatment as the receipt of an antimicrobial drug, chemotherapy, wound care, dialysis, or surgery in the 30 days before admission, and we defined a prehospital medical device as the presence of a urinary catheter, central line, mechanical ventilation, gastrostomy or jejunostomy tube, or tracheostomy in the 30 days before admission. We collected information regarding whether the patient had an outpatient medical encounter in the 7 days before hospital admission, including the date of the visit and the type of setting (eg, urgent care or medical subspecialty clinic). We defined health care exposure as the receipt of any prehospital medical treatment in the 30 days before admission, the presence of a prehospital medical device in the 30 days before admission, the occurrence of any outpatient medical encounter within 7 days of the sepsis diagnosis, or the occurrence of a stay of at least 2 days at a health care facility in the 30 days before admission. We determined the presence of systemic inflammatory response syndrome based on clinical information from the date of the sepsis diagnosis.^7^

Infections that were documented in the medical record as being present within 7 calendar days before or after the sepsis diagnosis and noted to be associated with sepsis in the discharge summary were included in this analysis. We defined organ dysfunction as any of the following documented in the medical record within 7 calendar days before or after sepsis diagnosis: receipt of invasive mechanical ventilation or noninvasive positive pressure ventilation, receipt of vasopressor medications (ie, norepinephrine, epinephrine, dopamine, phenylephrine, and vasopressin), systolic blood pressure less than 90 mm Hg, or altered mental status. We defined septic shock as 2 or more criteria of systemic inflammatory response syndrome plus the receipt of a vasopressor medication within 1 calendar day of sepsis diagnosis.

#### Pathogen Data and Death Certificates

Culture and culture-independent diagnostic test information was collected for each patient. We developed an algorithm based on specimen, diagnostic test, and organism type to identify organisms that were likely to be sepsis-associated pathogens (eTable 1 in the Supplement). Antimicrobial susceptibility testing results were collected for selected bacteria (*Enterococcus faecium, Enterococcus faecalis, Enterobacter* species, *Klebsiella pneumoniae*, *Escherichia coli, Pseudomonas aeruginosa, Staphylococcus aureus,* and *Streptococcus pneumoniae*) to determine the percentage of antimicrobial-resistant organisms. We collectively defined antimicrobial-resistant organisms as vancomycin-resistant *Enterococcus* species; carbapenem-resistant *Enterobacter* species, *K pneumoniae*, *E coli*, and *Pseudomonas aeruginosa*; methicillin-resistant *S aureus*; and penicillin-resistant *S pneumoniae*.

The EIP staff members obtained death certificate information from state vital statistics registries, including date of death, location of death, and underlying and immediate cause of death for patients who died during hospitalization or within 90 days of hospital discharge.

### Statistical Analysis

We performed descriptive analyses of demographic and clinical variables stratified by age. We used logistic regression analysis of complete cases with stepwise selection (entry and stay *P* values were set to .20 and .05, respectively) to assess risk factors associated with mortality within 30 days after sepsis diagnosis. Model selection included age group, septic shock status, presence of a sepsis-associated pathogen, and organ dysfunction within 7 days of sepsis diagnosis as a priori patient-level risk adjustment factors. Multicollinearity was ruled out using variance inflation factors. Analyses were performed using SAS software, version 9.4 (SAS Institute). Data were analyzed between May 1, 2018, and January 31, 2019.

## Results

We collected information from 28 individual hospitals across 10 EIP sites, with a median of 37 patients (interquartile range [IQR], 25-50 patients) included from each hospital. We excluded 22 patients with medical records that did not include clinical documentation of sepsis or an associated term.

### Patient Characteristics

Among 1078 adult patients (569 men [52.8%]), the median age was 64 years (IQR, 53-75 years) (Table 1). Most patients in all age groups had 1 or more underlying comorbidity, ranging from 141 patients (83.9%) aged 18 to 44 years to 369 patients (95.3%) aged 65 to 84 years. Nearly half of patients (515 patients [47.8%]) had Medicare insurance, 259 patients (24.0%) had Medicaid insurance, and 226 patients (21.0%) had private insurance as the primary payer. Most patients (806 patients [74.8%]) with sepsis or septic shock were admitted from a private residence, and 148 patients (13.7%) were admitted from a nursing home (Table 1). A total of 973 patients (90.3%) had community-onset sepsis. For approximately half of all patients (533 patients [49.4%]), the first documentation of sepsis in the medical record occurred in the emergency department, with the intensive care unit as the second most common setting.

**Table 1.** Characteristics of Patients With Sepsis and Septic Shock

Characteristic	No. (%),				
	Age group, y				Total
	18-44	45-64	65-84	≥85	
Patients, No.	**168**	**389**	**387**	**134**	**1078**
Male	86 (51.2)	214 (55.0)	219 (56.6)	50 (37.3)	569 (52.8)
Race					
White	85 (50.6)	251 (64.5)	254 (65.6)	99 (73.9)	689 (63.9)
Black or African American	31 (18.5)	80 (20.6)	78 (20.2)	19 (14.2)	208 (19.3)
American Indian or Alaskan native	10 (6.0)	11 (2.8)	6 (1.6)	2 (1.5)	29 (2.7)
Asian	7 (4.2)	9 (2.3)	10 (2.6)	7 (5.2)	33 (3.1)
Hispanic or Latino	31 (18.5)	47 (12.1)	35 (9.0)	12 (9.0)	125 (11.6)
Other^a^	15 (8.9)	15 (3.9)	9 (2.3)	4 (3.0)	43 (4.0)
Unknown	19 (11.3)	23 (5.9)	31 (8.0)	3 (2.2)	76 (7.1)
Underlying condition					
Any	141 (83.9)	366 (94.1)	369 (95.3)	123 (91.8)	999 (92.7)
Alcohol use	28 (16.7)	63 (16.2)	23 (5.9)	3 (2.2)	117 (10.9)
Diabetes (with or without complications)	37 (22.0)	140 (36.0)	166 (42.9)	44 (32.8)	387 (35.9)
Intravenous drug use	25 (14.9)	24 (6.2)	1 (0.3)	0 (0.0)	50 (4.6)
Immunosuppression^b^	32 (19.0)	98 (25.2)	131 (33.9)	23 (17.2)	284 (26.3)
Pulmonary disease^c^	20 (11.9)	100 (25.7)	113 (29.2)	31 (23.1)	264 (24.5)
Vascular disease^d^	10 (6.0)	92 (23.7)	154 (39.8)	70 (52.2)	326 (30.2)
Chronic kidney disease	15 (8.9)	54 (13.9)	96 (24.8)	37 (27.6)	202 (18.7)
Smoking	50 (29.8)	119 (30.6)	54 (14.0)	5 (3.7)	228 (21.2)
Location 4 d before hospital admission					
Private residence	132 (78.6)	296 (76.1)	279 (72.1)	99 (73.9)	806 (74.8)
Nursing home	7 (4.2)	33 (8.5)	78 (20.2)	30 (22.4)	148 (13.7)
Other acute care hospital or long-term acute care hospital	16 (9.5)	31 (8.0)	22 (5.7)	4 (3.0)	73 (6.8)
Other (incarcerated or homeless)	13 (7.7)	22 (5.7)	5 (1.3)	0	40 (3.7)
Unknown	0	7 (1.8)	3 (0.8)	0	11 (1.0)
Influenza vaccine in previous year	29 (17.3)	103 (26.5)	139 (35.9)	49 (36.6)	320 (29.7)
Pneumococcal vaccine any time before hospital admission^e^	31 (18.5)	125 (32.1)	177 (45.7)	64 (47.8)	397 (36.8)
Length of stay, median (IQR), d	8.0 (3.0-17.5)	8.0 (4.0-16.0)	8.0 (4.0-13.0)	6.0 (4.0-9.0)	7.0 (4.0-14.0)
ICU stay during sepsis hospitalization	116 (69.0)	281 (72.2)	271 (70.0)	71 (53.0)	739 (68.6)
Location of patient when sepsis first documented					
Emergency department	71 (42.3)	190 (48.8)	192 (49.6)	80 (59.7)	533 (49.4)
Inpatient ward	39 (23.2)	69 (17.7)	91 (23.5)	31 (23.1)	230 (21.3)
ICU	55 (32.7)	126 (32.4)	102 (26.4)	22 (16.4)	305 (28.3)
Other or unknown	3 (1.8)	4 (1.0)	2 (0.5)	1 (0.7)	10 (0.9)
Organ dysfunction within 7 d of sepsis diagnosis					
Any	113 (67.3)	319 (82.0)	329 (85.0)	112 (83.6)	873 (81.0)
Mechanical ventilation	52 (31.0)	157 (40.4)	118 (30.5)	28 (20.9)	355 (32.9)
Noninvasive positive pressure ventilation	22 (13.1)	67 (17.2)	74 (19.1)	25 (18.7)	188 (17.4)
Vasopressor initiated^f^	65 (38.7)	197 (50.6)	174 (45.0)	48 (35.8)	484 (44.9)
Systolic blood pressure level <90 mm Hg	92 (54.8)	239 (61.4)	239 (61.8)	83 (61.9)	653 (60.6)
Altered mental status	48 (28.6)	171 (44.0)	183 (47.3)	86 (64.2)	488 (45.3)
Creatinine level ≥2 mg/dL at any point during hospitalization^g^	41 (24.4)	158 (40.6)	150 (38.8)	42 (70.1)	391 (36.3)
Systemic inflammatory response syndrome					
Did not meet criteria	10 (6.0)	21 (5.4)	24 (6.2)	4 (3.0)	59 (5.5)
Met ≥2 criteria	111 (66.1)	220 (56.6)	230 (59.4)	91 (67.9)	652 (60.5)
Septic shock^h^	47 (28.0)	147 (37.8)	133 (34.4)	39 (29.1)	366 (34.0)
Death after sepsis diagnosis					
Within 5 d	14 (8.3)	52 (13.4)	50 (12.9)	26 (19.4)	142 (13.2)
Within 30 d	24 (14.3)	99 (25.4)	109 (28.2)	53 (39.6)	285 (26.4)
Within 90 d	29 (17.3)	116 (29.8)	130 (33.6)	68 (50.7)	343 (31.8)
Discharge location					
Private residence	106 (63.1)	167 (42.9)	141 (36.4)	34 (25.4)	448 (41.6)
Skilled nursing facility	13 (7.7)	70 (18.0)	118 (30.5)	47 (35.1)	248 (23.0)
Other acute care hospital or long-term acute care hospital	11 (6.5)	33 (8.5)	25 (6.5)	8 (6.0)	77 (7.1)
Other^i^	15 (8.9)	29 (7.5)	22 (5.7)	5 (3.7)	71 (6.6)

Abbreviations: ICU, intensive care unit; IQR, interquartile range.

^a^ Includes Native Hawaiian, Pacific Islander, and other races.

^b^ Includes steroid or immunosuppressive therapy, AIDS, hematologic malignancy, immunodeficiency or primary immunodeficiency, neutropenia, solid organ tumor with and without metastasis, history of bone marrow transplant, and history of solid organ transplant.

^c^ Includes chronic obstructive pulmonary disease and asthma.

^d^ Includes chronic heart failure, history of myocardial infarction, peripheral vascular disease, and stroke.

^e^ Includes conjugate or polysaccharide pneumococcal vaccines.

^f^ Includes norepinephrine, epinephrine, dopamine, phenylephrine, and vasopressin.

^g^ Excludes patients receiving dialysis.

^h^ Defined as 2 or more criteria of systemic inflammatory response syndrome plus the receipt of a vasopressor medication within 1 calendar day of sepsis diagnosis.

^i^ Includes homeless and incarcerated persons.

In total, 873 patients (81.0%) had evidence of organ dysfunction documented, and 366 patients (34.0%) met the criteria for septic shock. Across all age groups, lower respiratory tract infections (242 patients [22.4%]) were the most common factor underlying sepsis, followed by urinary tract infections (144 patients [13.4%]). A total of 130 patients (12.1%) had more than 1 sepsis-associated infection documented. However, 317 patients (29.4%) did not have a specific sepsis-associated infection documented in the discharge summary.

### Health Care Exposures

In total, 654 patients (60.7%) had at least 1 health care exposure before their hospital admission for sepsis (Table 2), and 260 patients (24.1%) had an outpatient medical encounter in the 7 days before hospital admission. Visits to a primary care physician or other outpatient medical specialist were most frequent among patients 65 years and older (38 of 119 patients [31.9%]), while emergency department and urgent care visits were most common among patients 64 years and younger (58 of 141 patients [41.1%]).

**Table 2.** Previous Health Care Exposures Among Patients With Sepsis and Septic Shock

Exposure	No. (%)				
	Age group, y				Total
	18-44	45-64	65-84	≥85	
Patients, No.	**168**	**389**	**387**	**134**	**1078**
Any health care exposure before hospital admission^a^	99 (58.9)	220 (56.6)	256 (66.1)	79 (59.0)	654 (60.7)
Prehospital medical treatment 30 d before admission					
Any treatment	74 (44.0)	153 (39.3)	177 (45.7)	43 (32.1)	447 (41.5)
Antimicrobial drugs	60 (35.7)	112 (28.8)	119 (30.7)	37 (27.6)	328 (30.4)
Chemotherapy	7 (4.2)	23 (5.9)	22 (5.7)	1 (0.7)	53 (4.9)
Wound care	10 (6.0)	24 (6.2)	37 (9.6)	4 (3.0)	75 (7.0)
Chronic dialysis	11 (6.5)	22 (5.7)	19 (4.9)	6 (4.5)	58 (5.4)
Surgery	8 (4.8)	13 (3.3)	27 (7.0)	6 (4.5)	54 (5.0)
Presence of medical device 30 d before admission					
Any device	42 (25.0)	74 (19.0)	90 (23.3)	13 (9.7)	219 (20.3)
Urinary catheter	17 (10.1)	31 (8.0)	45 (11.6)	11 (8.2)	104 (9.6)
Central line	30 (17.9)	44 (11.3)	48 (12.4)	1 (0.7)	123 (11.4)
Mechanical ventilator	6 (3.6)	9 (2.3)	8 (2.1)	1 (0.7)	24 (2.2)
G-tube, J-tube, or PEG-tube	10 (6.0)	13 (3.3)	9 (2.3)	1 (0.7)	33 (3.1)
Tracheostomy	6 (3.6)	4 (1.0)	7 (1.8)	1 (0.7)	18 (1.7)
Outpatient medical encounter within 7 d of hospital admission					
Any encounter	43 (25.6)	98 (25.2)	92 (23.8)	27 (20.1)	260 (24.1)
Medical or pediatric specialty	12 (7.1)	26 (6.7)	31 (8.0)	6 (4.5)	75 (7.0)
Emergency department or urgent care	24 (14.3)	34 (8.7)	20 (5.2)	2 (1.5)	80 (7.4)
Primary care	4 (2.4)	27 (6.9)	21 (5.4)	17 (12.7)	69 (6.4)
Stayed ≥2 d at health care facility in previous 30 d					
Hospital	41 (24.4)	93 (23.9)	97 (25.1)	27 (20.1)	258 (23.9)
Nursing home	5 (3.0)	31 (8.0)	70 (18.1)	31 (23.1)	137 (12.7)
Inpatient rehabilitation	3 (1.8)	1 (0.3)	1 (0.3)	1 (0.7)	6 (0.6)
Other or unknown	6 (3.6)	21 (5.4)	17 (4.4)	7 (5.2)	51 (4.7)

Abbreviation: G-tube, gastrostomy tube; J-tube, jejunostomy tube; PEG-tube, percutaneous endoscopic gastrostomy tube.

^a^ Includes any medical treatment in the 30 days before hospital admission, the presence of a medical device in the 30 days before hospital admission, the occurrence of any outpatient medical encounter within 7 days of the sepsis diagnosis, and the occurrence of a stay of 2 or more days at a health care facility in the previous 30 days.

A total of 447 patients (41.5%) received prehospital medical treatment in the 30 days before hospitalization; of those, the largest proportion were patients who received antibiotic medications in the 30 days before sepsis hospitalization (328 patients [73.4%]). A total of 219 patients (20.3%) had a medical device present in the 30 days before sepsis hospitalization, and 355 patients (32.9%) stayed overnight in a health care facility at some point in the 30 days before hospital admission.

### Sepsis-Associated Pathogens

A total of 1069 patients (99.2%) had at least 1 bacterial culture drawn. Blood cultures (1013 patients [94.0%]) were most common, followed by urine (663 patients [61.5%]) and lower respiratory (310 patients [28.8%]) cultures. A sepsis-associated pathogen was identified in 613 patients (56.9%) (Table 3) and was most commonly found in blood cultures (290 patients [47.3%]).

**Table 3.** Common Organisms in Patients With Sepsis and Septic Shock^a^

Organism	Patients with organism, No. (%) (N = 1078)^a^
Any type or name	613 (56.9)
**Gram-positive**	
*Staphylococcus* spp	135 (12.5)
*Staphylococcus aureus*	121 (11.2)
*Streptococcus* spp	99 (9.2)
*Streptococcus pneumoniae*	37 (3.4)
*Streptococcu*s, viridans group	25 (2.3)
Group A streptococcus	14 (1.3)
*Enterococcus* spp	45 (4.2)
*Enterococcus faecalis*	21 (1.9)
*Enterococcus faecium*	11 (1.0)
*Clostridium* spp	58 (5.4)
*Clostridioides difficile*	53 (4.9)
**Gram-negative**	
*Escherichia coli*	149 (13.8)
*Klebsiella* spp	65 (6.0)
*Klebsiella pneumoniae*	56 (5.2)
*Pseudomonas* spp	47 (4.4)
*Pseudomonas aeruginosa*	42 (3.9)
*Proteus* spp	33 (3.1)
*Proteus mirabilis*	29 (2.7)
*Bacteroides* spp	13 (1.2)
*Enterobacter* spp	12 (1.1)
*Citrobacter* spp	11 (1.0)
**Virus**	
Influenza	24 (2.2)
Rhinovirus	13 (1.2)
**Fungus**	
*Candida* spp	31 (5.1)

Abbreviation: spp, several species.

^a^ If an organism was documented in the medical record, it was classified as a sepsis-associated pathogen using an algorithm based on the site of collection and specimen type, type of organism, and testing method (eTable 1 in the Supplement).

The most common sepsis-associated pathogens from any culture site were *E coli* (149 patients [13.8%]), *S aureus* (121 patients [11.2%]), *K pneumonia*e (56 patients [5.2%]), and *Clostridioides difficile* (53 patients [4.9%]). Among the 613 patients with a sepsis-associated bacterial pathogen identified, 332 patients (54.2%) had antimicrobial susceptibility test results reported in the medical record. Of those, 72 patients (21.7%) had an antimicrobial-resistant pathogen; the most common antimicrobial-resistant sepsis-associated pathogen was methicillin-resistant *S aureus* (46 patients [63.9%]). Among 166 patients with sepsis associated with Enterobacteriaceae or *Pseudomonas aeruginosa* for which antimicrobial susceptibility data were available, 12 patients (7.2%) had resistance to carbapenem medications.

### Factors Associated With Death

Overall, 343 patients (31.8%) died within 90 days of the date of sepsis diagnosis. A total of 142 patients (13.2%) died within 5 days of diagnosis; of those, 51 patients (35.9%) died within 1 day of sepsis diagnosis. A total of 143 patients (13.3%) died between 6 and 30 days after sepsis diagnosis, and 58 patients (5.4%) died between 31 and 90 days after diagnosis. The final multivariable model of factors associated with 30-day mortality is shown in Table 4; all variables assessed in the model are listed in eTable 2 in the Supplement.

**Table 4.** Risk Factors Associated With Mortality at 30 Days After Sepsis Diagnosis^a^

Variable^b^	Total patients, No. (N = 1061)	Deaths, No.	Odds ratio (95% CI)	*P* value
Age group, y				
18-44	166	24	1 [Reference]	NA
45-64	378	96	1.64 (0.96-2.80)	.07
65-84	384	109	1.96 (1.14-3.39)	.02
≥85	133	53	4.68 (2.51-8.76)	<.001
Sepsis-associated pathogen identified				
Yes	605	148	1.21 (0.87-1.68)	.27
No	456	134	1 [Reference]	NA
Organ dysfunction^c^				
Yes	879	263	2.33 (1.35-4.04)	.003
No	182	19	1 [Reference]	NA
Septic shock^d^				
Yes	362	148	2.65 (1.93-3.65)	<.001
No	699	134	1 [Reference]	NA
Receipt of influenza or pneumococcal vaccine				
Yes	493	124	1 [Reference]	NA
No	568	158	1.51 (1.10-2.06)	.01
Immunosuppression^e^				
Yes	280	111	2.52 (1.81-3.52)	<.001
No	781	171	1 [Reference]	NA
Cirrhosis				
Yes	64	32	3.59 (2.03-6.32)	<.001
No	997	250	1 [Reference]	NA
Underlying vascular disease				
Yes	323	109	1.54 (1.10-2.15)	.01
No	738	173	1 [Reference]	NA
Sepsis-associated infection documented at hospital discharge				
No infection	317	102	1 [Reference]	NA
>1 infection	130	34	0.87 (0.52-1.46)	.61
Infection with source undetermined	28	12	1.62 (0.67-3.91)	.28
Type of infection				
Abdominal^f^	74	18	0.63 (0.34-1.19)	.15
Bloodstream	58	21	1.57 (0.80-3.09)	.18
Respiratory^g^	239	62	0.81 (0.54-1.21)	.30
Skin and soft tissue	60	8	0.49 (0.21-1.14)	.10
Urinary tract	141	19	0.39 (0.22-0.71)	.002
Other^h^	14	6	3.28 (0.98-11.02)	.05

Abbreviation: NA, not applicable.

^a^ Variable descriptions and reference levels are listed in eTable 2 in the Supplement.

^b^ Age group, septic shock, sepsis-associated pathogen, and presence of organ dysfunction were included in the model. A full list of variables is in eTable 2 in the Supplement.

^c^ Includes any of the following documented in the medical record within 7 calendar days of initial sepsis documentation: receipt of invasive mechanical ventilation or noninvasive positive pressure ventilation, receipt of vasopressor medication, systolic blood pressure level less than 90 mm Hg, or altered mental status.

^d^ Defined as 2 or more criteria of systemic inflammatory response syndrome plus the receipt of a vasopressor medication within 1 calendar day of sepsis diagnosis.

^e^ Includes steroid or immunosuppressive therapy, AIDS, hematologic malignancy, immunodeficiency or primary immunodeficiency, neutropenia, solid organ tumor with and without metastasis, history of bone marrow transplant, and history of solid organ transplant.

^f^ Includes any intra-abdominal, gastrointestinal tract, or hepatobiliary infection.

^g^ Includes lower respiratory infection and pneumonia.

^h^ Includes bone and joint infections; cardiovascular infections; infections of the ear, nose, or throat; central nervous system infections; disseminated viral infections; and infections of the reproductive tract.

Patients who lacked medical record documentation of receiving either the influenza or pneumococcal vaccine had a significantly higher likelihood (odds ratio [OR], 1.51; 95% CI, 1.10-2.06) of dying within 30 days compared with patients who had documentation of receiving either vaccine. Patients who were immunosuppressed (OR, 2.52; 95% CI, 1.81-3.52), had cirrhosis (OR, 3.59; 95% CI, 2.03-6.32), or had underlying vascular disease (OR, 1.54; 95% CI, 1.10-2.15) also had a higher likelihood of dying within 30 days compared with patients without those conditions. Patients with a urinary tract infection had a lower likelihood (OR, 0.39; 95%, CI 0.22-0.71) of dying within 30 days compared with those without a documented infection.

## Discussion

Sepsis is an important public health challenge, and characterization of the disease course and health care exposures of patients with sepsis in the days or weeks before hospitalization may help to identify opportunities for improving outcomes. In this cohort of patients, which included in-depth medical record reviews of more than 1000 patients from diverse geographic areas, we observed that most adult patients with sepsis had outpatient or other health care facility exposures or medical treatment in the weeks before hospital admission for sepsis. These preadmission health care experiences may offer opportunities, such as practitioner and patient educational interventions, to alter the disease course of patients at risk of experiencing severe outcomes.

Educational interventions regarding sepsis identification and treatment have mainly focused on inpatient health care practitioners and acute care settings by using a universal approach to recognize signs and symptoms, without regard to the diversity of sepsis presentations that may occur based on patient age, underlying conditions, or previous health care interactions.^2,8^ Our data suggest that important opportunities are available to educate practitioners outside of the hospital to recognize the signs and symptoms of sepsis; these findings are similar to the results of previous studies.^3,9^ Liu et al^9^ reported that nearly half of patients with sepsis visited an outpatient clinician in the 7 days before they were hospitalized; nearly one-third of patients were assigned diagnosis codes for acute infection, and 20% to 40% of patients were prescribed antibiotic medications at the visit. Outpatient health care practitioners, including primary care physicians, medical subspecialists, and other health care clinicians, play an important role in identifying patients at high risk of developing sepsis who may benefit from close follow-up and can assist in recognizing and treating sepsis-associated infections before the onset of organ dysfunction. The Get Ahead of Sepsis campaign designed by the Centers for Disease Control and Prevention^10^ provides a variety of educational materials to help patients, caregivers, and different health care practitioners recognize the signs and symptoms of sepsis.

We identified factors associated with mortality in adult patients who were hospitalized with sepsis; in particular, we found that increasing age, the presence of organ dysfunction, and selected underlying conditions were associated with death. Of note, we observed that the lack of influenza or pneumococcal vaccination was also associated with mortality, although to a lesser extent than underlying conditions and severity of illness. Although these vaccinations offer protection against specific sepsis-associated pathogens, we hypothesize that vaccination status also serves as a surrogate for broader health care access and health maintenance practices that were unmeasured in our analysis.^11,12^

Several analyses have indicated that underlying conditions are associated with sepsis outcomes and may be as important to sepsis outcomes as hospital care. Hatfield et al^4^ reported that markers of health status are associated with sepsis mortality even after accounting for in-hospital care, suggesting that efforts that encourage patients to seek care before the onset of organ failure could be associated with reductions in sepsis mortality. Rhee et al^13^ reviewed the medical records of 198 deceased patients with sepsis to assess the extent to which these deaths were preventable. They found that 23% of the cohort experienced some level of suboptimal care but considered only 12% of deaths to be potentially preventable, suggesting that patient factors, such as comorbidities and baseline health status, play an important role in sepsis outcomes. Our analysis also highlights the association of underlying comorbidities and severity of illness with sepsis mortality, suggesting that sepsis education and preemptive care may be particularly important for specific patient populations or health care practitioners. The use of risk stratification models for outpatients who present with acute infections could improve the identification of patients with the greatest risk of disease progression who may benefit from closer monitoring.^14^

Infection source control and the identification of sepsis-associated pathogens are important components of sepsis treatment that can guide the selection and treatment duration of antimicrobial drugs.^2^ Even in our population of patients, who were identified through the use of explicit administrative codes for severe sepsis and septic shock, nearly 40% of patients did not have a sepsis-associated pathogen identified in our analysis; 30% of patients had no documented sepsis-associated infection on their discharge summary, despite having a billing code for severe sepsis or septic shock. It is possible that previous antibiotic medication exposure (identified in 30% of the patients in our analysis) was a factor in the low percentage of patients for whom a sepsis-associated pathogen was identified. In addition, we did not find an association between the identification of a sepsis-associated pathogen and mortality within 30 days after sepsis diagnosis, which could be owing to the prevalence of broad-spectrum antimicrobial treatment among patients with suspected sepsis, regardless of whether a sepsis-associated pathogen had been identified. Better diagnostic tests for sepsis and infectious diseases are needed to quickly and accurately identify patients with sepsis and infections associated with specific pathogens and to improve antimicrobial drug use and minimize antimicrobial-associated risks.^15,16^

### Limitations

This study has several limitations. Patients with sepsis and septic shock were identified through the use of administrative codes along with confirmation that at least 1 health care practitioner had documented sepsis in the patient’s medical record rather than through the application of an objective definition based on physiological or laboratory criteria. Explicit codes for severe sepsis and septic shock are specific but insensitive,^1^ and we may have captured more severe cases but missed cases of sepsis that were less severe or less easily diagnosed. We only considered an infection to be associated with sepsis or septic shock if it was documented in the discharge summary as associated with sepsis; we may have misclassified infection types among patients or included patients who may not have had sepsis or septic shock.

Because this analysis relied on data abstraction from hospital medical records, the data obtained might be incomplete. Information documented during outpatient clinic visits or nursing home stays, on receipt of home health care services or vaccines, or on indication for prehospital antibiotic medications may have been unavailable. We also limited the period before sepsis hospitalization during which these factors were assessed. In particular, the proportion of patients with health care exposures, including outpatient visits, prehospital medical treatment, and prehospital medical devices, could have been underestimated.

We used an algorithm (eTable 1 in the Supplement) to identify sepsis-associated pathogens based on available clinical information; organisms could have been misidentified as sepsis-associated pathogens. Furthermore, we included a limited number of clinical factors to define organ dysfunction (Table 1); we excluded hepatic dysfunction, thrombocytopenia, and kidney dysfunction because it was difficult to determine the true baseline values for each patient and to identify whether an abnormal laboratory value was associated with sepsis or septic shock. We only included patients with sepsis or septic shock in our analysis, and we cannot draw conclusions about the likelihood that risk factors are associated with the onset of sepsis or septic shock.

## Conclusions

Our data indicate that, across all age groups, most adult patients with sepsis have chronic underlying illnesses, and a substantial percentage have prehospital opportunities for care that could be used to disrupt the progression from infection to sepsis, hospital admission and, in almost one-third of cases, death. Public health and medical professionals can work to ensure that sepsis educational initiatives reach a wide array of outpatient health care settings and practitioners as well as patients and hospital-based practitioners to raise awareness of sepsis as an important public health problem.

## Notes

Supplement. **eTable 1.** Determining the Causative Pathogen: Specimen Type, Test Type, and Criteria **eTable 2.** Variables Included in the Multivariable Model Assessing Risk Factors Associated With Mortality at 30 Days After Sepsis Diagnosis
