# Supplementary material for: Assessment of Health Care Exposures and Outcomes in Adult Patients With Sepsis and Septic Shock
Source: JAMA Netw Open. 2020 Jul 7;3(7):e206004. doi: 10.1001/jamanetworkopen.2020.6004 (PMC7341174; doi:10.1001/jamanetworkopen.2020.6004)
Supplement: Supplement. — eTable 1. Determining the Causative Pathogen: Specimen Type, Test Type, and Criteria eTable 2. Variables Included in the Multivariable Model Assessing Risk Factors Associated With Mortality at 30 Days After Sepsis Diagnosis [file jamanetwopen-3-e206004-s001.pdf]

Supplementary Online Content

Fay K, Sapiano MRP, Gokhale R, et al. Assessment of health care exposures and outcomes in adult patients with sepsis and septic shock. *JAMA Netw Open*. 2020;3(7):e206004. doi:10.1001/jamanetworkopen.2020.6004

eTable 1. Determining the Causative Pathogen: Specimen Type, Test Type, and Criteria

eTable 2. Variables Included in the Multivariable Model Assessing Risk Factors Associated With Mortality at 30 Days After Sepsis Diagnosis

This supplementary material has been provided by the authors to give readers additional information about their work.

© 2020 Fay K et al. *JAMA Network Open*.

eTable 1. Determining the Causative Pathogen: Specimen Type, Test Type, and Criteria

Specimen Type	Test Type	Criteria to be included as a causative pathogen	Criteria to determine organism is not a causative pathogen
**Blood**	**Positive Microbiologic Culture**	All bacteria, viruses, fungi	Common commensals[1]
	**Non-culture Diagnostic Test**	All bacteria, fungi, parasites, viruses	
**Cerebral Spinal Fluid**	**Positive Microbiologic Culture**	All bacteria, viruses, fungi, parasites	
	**Non-culture Diagnostic Test**		
**Lower Respiratory Specimen**	**Positive Microbiologic Culture**	-- All viruses and bacteria if lower respiratory or pneumonia infection indicated<br>-- Included certain fungi (*Cryptococcus* and *Cladosporium*) regardless of whether infection documented	-- Common commensals[2]<br>-- All viruses/bacteria/fungi if lower respiratory or pneumonia infection not documented
	**Non-culture Diagnostic Test**	All viruses	*Candida* spp or other yeast
**Pleural fluid**	**Positive Microbiologic Culture**	Include all bacteria, viruses, fungi	
**Stool**	**Positive Microbiologic Culture**	-- All parasites<br>-- Bacteria, fungi, viruses based on documentation of clinical infection	

© 2020 Fay K et al. *JAMA Network Open*.

	**Non-culture Diagnostic Test**	*Clostridium* spp. and viruses	
**Urine**	**Positive Microbiologic Culture**	-- Bacteria/fungi if urinary tract infection was documented as an infection<br>-- All parasites (*Echinococcus*)	Bacteria/fungi without documented urinary tract infection
	**Non-culture Diagnostic Test**	-- Include Legionella/*Streptococcus pneumoniae* antigen test positive results	Yeast (i.e. beta-d-glucan), certain viruses if no clinical information
**Wound**	**Positive Microbiologic Culture**	All bacteria/fungi/viruses if skin and soft tissue infection indicated and/or bone and joint infection documented	Bacteria/fungi/viruses if no infection documented
	**Non-culture Diagnostic Test**	All fungi, bacteria, viruses only if patient has skin or soft tissue infection or bone and joint infection	Bacteria/viruses/fungi if no infection documented
**Other Specimen[3]**	**Positive Microbiologic Culture**	All bacteria, fungi or viruses isolate from cultures of normally sterile sites	
	**Non-culture Diagnostic Test**	--All bacteria from a sterile site<br>--Certain bacteria/fungi/viruses from non-sterile sites depending on site and clinical infection	Bacteria/fungi/viruses if no corresponding infection indicated in the medical chart

[1]Based on National Healthcare Safety Network (NHSN) criteria for common commensals.
[2]*Candida* spp, coagulase-negative *Staphylococcus* species, *Enterococcus* species.
[3]Sterile sites: abdominal abscess, abdominal fluid, abdominal tissue, abscess, blood, bone, brain, eye, maxillary fluid, mediastinum fluid, heart tissue, joint tissue, kidney tissue, lung tissue, lymph nodes, peritoneal fluid, pelvic fluid, pleural biopsy fluid, plasma, serum, shoulder tissue, shut fluid, sinus fluid, brain tissue or other general tissue, thigh fascia, fluid from a thoracentesis, vitreous tissue, muscle . Non-sterile sites: skin abscess, anal swab, bronchalverolar lavage fluid, catheter tips, cervical swab, cheek tissue, chest tube, endotracheal tube, esophageal tissue, eye tissue, fluid (unknown location), gallbladder tissue, cervical swab, groin swab, stomach tissue, incision wound, nephrostomy, nasal swabs, mouth, skin swabs, catheter swabs, vaginal culture, skin, bile.

© 2020 Fay K et al. *JAMA Network Open*.

eTable 2. Variables Included in the Multivariable Model Assessing Risk Factors Associated With Mortality at 30 Days After Sepsis Diagnosis

Variable	Levels	Reference level	Values
Gender	2	Male	Female; Male
Age group	4	18-44 years old	18-44 years old; 45-64 years old; 65-84 years old; 85 years and older
Race	3	White	Black; White; Not black or white
Diabetes	2	No	Yes, No
Immunosuppressed	2	No	Yes, No
Vascular Disease	2	No	Yes, No
Pulmonary Disease	2	No	Yes, No
Alcohol Abuse	2	No	Yes, No
Smoking	2	No	Yes, No
Intravenous Drug Use	2	No	Yes, No
Cirrhosis	2	No	Yes, No
Chronic Dialysis	2	No	Yes, No
Organ dysfunction within 7 days	2	No organ dysfunction	No organ dysfunction; Organ dysfunction within 7 days
Septic Shock	2	No shock	No shock; Shock
Any medical treatment in prior 30 days	2	None	Any treatment in prior 30 days; None
Antimicrobial therapy in prior 30 days	2	Antimicrobial therapy	Antimicrobial therapy; None
Device use in prior 30 days	2	Any device in prior 30 days	Any device in prior 30 days; None
Documented infections	8	No infection documented on discharge as cause of sepsis	Abdominal; More than one infections documented on discharge as cause of sepsis; No infection documented on discharge as cause of sepsis; Other; Respiratory; Skin and Soft Tissue; Undetermined; Urinary
Causative pathogen	2	No pathogen	No pathogen; Any pathogen
Location 4 days prior to sepsis diagnosis	4	Private residence	Private residence; Nursing home/skilled nursing facility; Long term acute care hospital or other acute care hospital; Other
Community or hospital onset	2	Community onset	Community onset; Hospital onset
Outpatient medical encounter	2	None	Any Outpatient encounter; None
Influenza or pneumococal vaccine	2	Receipt of either vaccine	Influenza or pneumococal vaccine; Neither

© 2020 Fay K et al. *JAMA Network Open*.
